# Influence of relative density on quasi-static and fatigue failure of lattice structures in Ti6Al4V produced by laser powder bed fusion

**DOI:** 10.1038/s41598-021-98631-3

**Published:** 2021-09-29

**Authors:** Markel Alaña, Antonio Cutolo, Sergio Ruiz de Galarreta, Brecht Van Hooreweder

**Affiliations:** 1 Department of Mechanical Engineering and Materials, Universidad de Navarra, TECNUN Escuela de Ingenieros, Paseo Manuel de Lardizabal, 13, 20018 San Sebastian, Spain; 2 KU Leuven Department of Mechanical Engineering, Celestijnenlaan 300, 3001 Leuven (Heverlee), Belgium; 3 Members Flanders Make, Leuven, Belgium

**Keywords:** Mechanical engineering, Structural materials

## Abstract

Lattice structures produced by additive manufacturing have been increasingly studied in recent years due to their potential to tailor prescribed mechanical properties. Their mechanical performances are influenced by several factors such as unit cell topology, parent material and relative density. In this study, static and dynamic behaviors of Ti6Al4V lattice structures were analyzed focusing on the criteria used to define the failure of lattices. A modified face-centered cubic (FCCm) lattice structure was designed to avoid the manufacturing problems that arise in the production of horizontal struts by laser powder bed fusion. The Gibson–Ashby curves of the FCCm lattice were obtained and it was found that relative density not only affects stiffness and strength of the structures, but also has important implications on the assumption of macroscopic yield criterion. Regarding fatigue properties, a stiffness based criterion was analyzed to improve the assessment of lattice structure failure in load bearing applications, and the influence of relative density on the stiffness evolution was studied. Apart from common normalization of S–N curves, a more accurate fatigue failure surface was developed, which is also compatible with stiffness based failure criteria. Finally, the effect of hot isostatic pressing in FCCm structures was also studied.

## Introduction

Additive manufacturing (AM), and laser powder bed fusion (LPBF) of metals specifically, enables the production of exceptionally complex parts in a cost-effective manner, including architected lattice structures^1,2^. These structures offer a set of mechanical property combinations unavailable until few decades ago. Lattice structures can be used to design metamaterials when analyzed at macroscopic level^3,4^. These metamaterials can be formed by a three dimensional pattern of a repeating unit cell (UC), or by stochastic arrangements of structural units that fill a certain space to form a part^5^.

The large amount of available configurations makes lattice structures interesting for diverse applications, ranging from structural components as well as in energy absorption, heat exchanger devices, vibration attenuators or for catalytic purposes^6–9^. In terms of load carrying applications, the freedom that AM offers regarding the manufacturability of geometries enables the design of lattice structures tailoring specific mechanical properties that meet specific needs^10,11^.

Lattice structures can be categorized in stretching or bending dominated structures depending on the configuration of their struts^12^. Along with the topology of the structures, relative density is an important driving factor of their mechanical properties, defined by the proportion of the parent material within a Representative Volume Element (RVE) of the lattice structure.

The influence of relative density on the quasi-static lattice mechanical properties has been studied for different structures like BCC^13^, diamond^14^, octet truss^15^, FCC^16^, rhombic dodecahedron^17^ or cubic^18^, among others^19^, covering a wide range of stiffness and strength levels. Yield strength or plateau stress are in turn used to better predict the fatigue properties of those structures, because they implicitly include the effect of variables such as material, microstructure, surface roughness, relative density or manufacturing deviations^18^. Ahmadi et al.^20^ concluded that the accuracy of this approach was highly dependent on the material and the unit cell topology. On the other hand, Van Hooreweder et al.^21^ developed a Local Stress Method (LSM) to predict the fatigue behavior of lattice structures based on a beam model, and considering only local tensile stresses. In addition, the effect of heat and surface treatments was also extensively studied, concluding that a combination of surface treatments like chemical etching or sand blasting with Hot Isostatic Pressing (HIP) sensibly improves the lattice structures fatigue life^21–23^.

Most of the experimental work in fatigue is conducted under uniaxial compression-compression loads due to the simplicity of the test configuration^24^, and as far as the authors know there is no standard failure criterion that defines the lattice specimen failure in fatigue tests. Nevertheless, it is common to consider the failure of a specimen when it loses most or all of its stiffness^23,25–27^, e.g. 90% of stiffness reduction, or permanent displacement drops^21,22,28^. These criteria imply the loss of the load carrying capacity of the specimen, yet in the case of parts integrating lattices for load bearing applications, the changes in stiffness or deformations on the lattice might lead to the increase of stresses in the part. Thus, it is useful to consider other criteria to determine the failure of lattice structures, as done by Boniotti et al.^29^, where a 10% stiffness loss failure criterion was used to analyze the fatigue of AlSi7Mg lattice structures.

Nonetheless, AM has some manufacturing constraints. In order to avoid complex post-processing, metal lattice structures are usually fabricated without structural support. This issue limits the geometry freedom, and to ensure strut quality, overhangs angles not lower than 45° are suggested. Horizontal struts produced by LPBF have an overall lower quality than inclined or vertical struts, with higher strut porosity levels and lower dimensional accuracy^30^. This has significant effects on the mechanical properties of lattice structures, and limits their capability to perform under certain load orientations^31^. Another constraint for LPBF structures that can deteriorate AM parts are residual stresses which are more pronounced when using self-supporting structures.

In this work, a modified FCC lattice structure (FCCm) was designed by removing the horizontal struts of the FCC unit cell. The main purpose of this work is to study the fatigue life of the Bravais-oriented FCCm lattice structure for different relative densities. In order to develop the fatigue life models, lattice structures of several relative densities were produced with LPBF Ti6Al4V. The fatigue behavior was studied for the different relative densities, analyzing the evolution of the stiffness of the specimens, and assessing a stiffness based failure criterion for fatigue. A new method to predict the fatigue life of lattice structures for a wide range of relative densities is also proposed, which can be used along with stiffness based failure criteria. Furthermore, the interaction between the mesoscopic stress state and macroscopic metamaterial properties were numerically investigated to explore in depth in the assumption of the 0.2% offset stress as the macroscopic yield strength of lattice structures. Finally, a batch of as-built FCCm lattices was also produced to analyze the effect of heat treatment on the quasi-static and fatigue properties of the FCCm structures.

## Materials and methods

### Design and production of lattice structures

**Figure 1:**
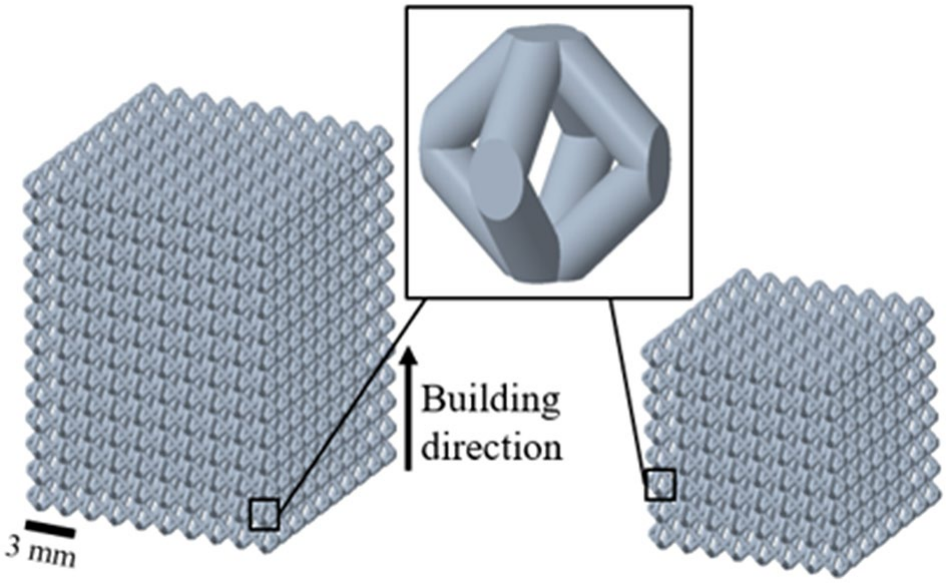
Specimen geometry for 20% relative density design for HIP (left) and as-built (right) conditions.

Lattice structures were designed based on the FCCm unit cell, which is formed by 6 nodes and 8 struts forming an angle of 45° with respect to the build plate as in Fig. 1. A unit cell size of 1.5mm was used, and structures of two different sizes were produced. Lattices of 10%, 20% and 30% relative densities—referred as RD01, RD02 and RD03—were designed using the formula developed in Alaña et al.^32^, with diameters of 0.24 mm, 0.36 mm and 0.46 mm respectively, and a width of 15 mm and height of 19.5 mm (left in Fig. 1). These structures were HIP treated at 920°C for two hours with a pressure of 1000 bar after production. On the other hand, structures of 12 mm size were also designed based in the same unit cell, and with a prescribed relative density of 20% (right in Fig. 1). These structures were not treated after production and are named AB (as-built). All the samples were produced in Ti6Al4V by LPBF on a Mlab machine (Concept Laser).

### Morphological characterization and mechanical testing

For each of the produced specimens, the relative density of the structures was measured by dry weighing. The total volume of the specimen was calculated from caliper measurements, and a theoretical density of 4.42 g/cm^3^ was assumed for Ti6Al4V. Furthermore, the strut density was also measured by means of Archimedes method, submerging the specimens in ethanol.

Quasi-static compression tests were carried out on an Instron 3360 with a 30 kN load cell. A crosshead velocity of 0.9 mm/min was used, and Teflon sheets of 0.2 mm were used to reduce the friction between the specimen and the compression plates. The strain was measured using the Instron Video Extensometer AVE2. For the RD03 structures an Instron 5982 was used with a 100 kN load cell due to their higher strength. In this case, the crosshead displacement was measured, and strain was calculated by compensating the compliance of the machine.

Load controlled compression-compression fatigue tests were performed on an Instron Electropuls E10000 machine, with a frequency of 15 Hz and a load ratio (R) of 10. Tests were stopped when collapse of the specimen occurred, or after reaching $10^6$ cycles. Furthermore, the stiffness of the samples was measured every 1000 cycles. After compensating the stiffness of the machine, an additional failure criterion was established at 10% stiffness loss of the sample. Due to the high strength of the RD03 specimens a Schenck equipped with a load cell of 160 kN was used for fatigue testing. The fatigue strength (FS) of the structures at $10^6$ cycles was obtained by means of the staircase method^33^. An arbitrary stress is applied to the specimen ($\sigma _{n}$), and after the prescribed number of cycles is reached ($N_{limit} = 10^6$) the stress level is increased and the same specimen is tested again. The FS is obtained by means of Eq. (1).

1 $$\begin{aligned} \sigma _{FS} = \sigma _{n-1} + \frac{N_{failure}}{N_{limit}} (\sigma _{n} - \sigma _{n-1}) \end{aligned}$$

### Numerical models

In order to understand the effect of the relative density on the macroscopic properties of the FCCm lattice a Finite Element (FE) analysis was carried out. The simulations were also performed to study the relationship between the lattice macroscopic properties and the bulk material as the relative density varies.

**Figure 2:**
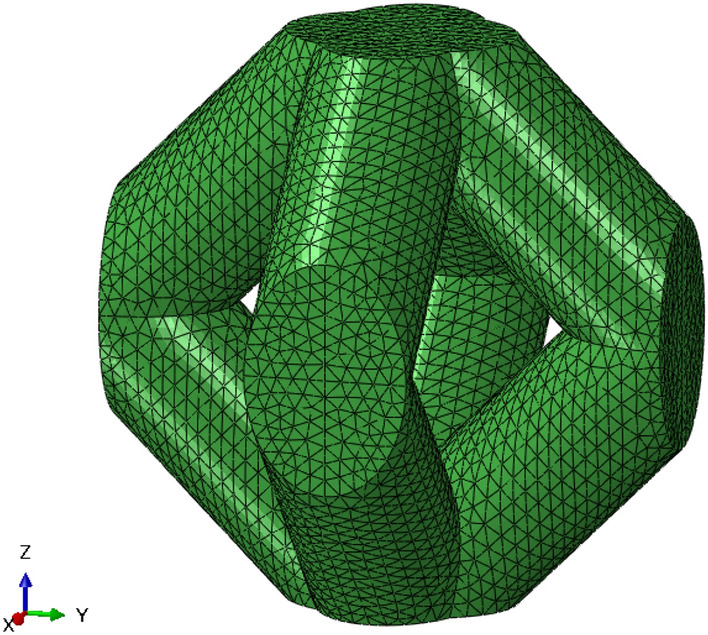
FE model of a unit cell of the RD02 specimen.

The FE models were constructed with a single unit cell (Fig. 2) with Periodic Boundary Conditions (PBC)^34^. On the one hand, FE models were built with relative densities corresponding to the values obtained by dry weighing, in order to compare directly with the experiments. Therefore, a part of the manufacturing deviation was already accounted for. On the other hand, several numerical models with relative densities between 5% and 40% were also designed in order to analyze the interaction between macroscopic and mesoscopic stress levels and their variability for different relative densities. Abaqus 2020 was used for the analyses, with second order tetrahedral elements (C3D10) after conducting a sensitivity analysis, and the element size was designed to be ten times smaller than the strut diameter for each model. Linear elasticity was assumed, and J2 plasticity was used to model plastic behavior. The material was assigned a Young’s modulus of 129 MPa, a Poisson ratio of 0.342 and a yield strength of 921 MPa.

### Normalization of fatigue curves

For each relative density the fatigue experimental data were used to derive specific S–N curves. These curves were normalized with the global 0.2% offset stress obtained from the quasi-static compression tests to assess the variability of the normalized curves for different relative densities and to analyze the effect of the HIP.

Moreover, the Local Stress Method (LSM) developed by Van Hooreweder et al.^21^ was implemented in the FCCm structures. This normalization is based on a Timoshenko beam model of the struts, and considers only the tensile stress of the beam model to assess the fatigue life of the structures. In order to apply the LSM for a wide range of relative densities the method was modified to consider the variability of strut length ($l_{strut}$) apart from diameter (*d*) changes. Thus, this equation gives the analytical tensile stress across the FCCm struts:

2 $$\begin{aligned} \sigma _{LSM} = F \left( \frac{16 l_{strut} \cos \theta }{\pi d^3} - \frac{4 \sin \theta }{\pi d^2} \right) \end{aligned}$$

**Figure 3:**
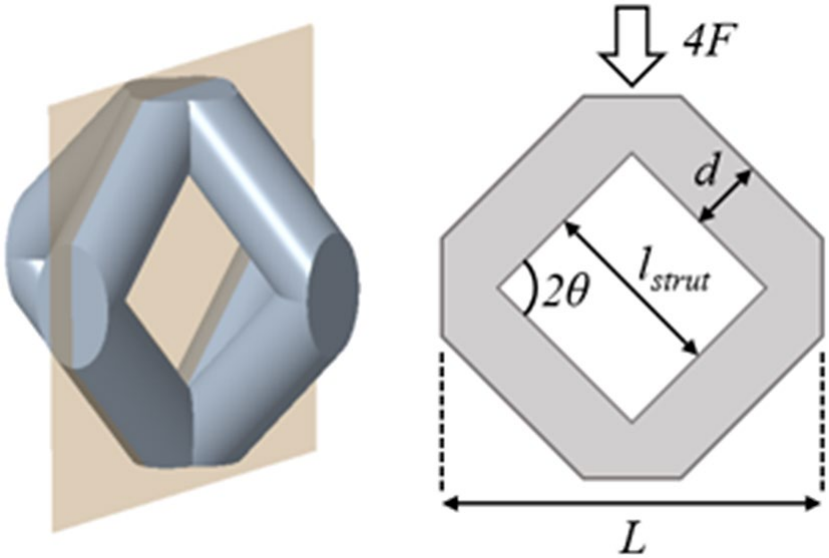
Parameters to obtain the maximum tensile stress in each strut by using the Local Stress Method.

In which *F* is force applied on the single strut and $\theta$ is the angle between the strut axis and the horizontal plane, as shown in Fig. 3. For FCCm structures $\theta =45^{\circ }$. The relation between the strut diameter and the relative density for FCCm structures is given by Eq. (3), where *L* represents unit cell size.

3 $$\begin{aligned} \rho _{rel} = \alpha \left( \frac{d}{L}\right) ^3 + \beta \left( \frac{d}{L}\right) ^2 \text {,} \; \; \; \text {with} \; \alpha = -3.91 \text { and } \beta = 4.44 \end{aligned}$$

### Fatigue failure surface

The S–N curves were used to define a S–N–$\rho _{rel}$ surface, which expresses the fatigue properties of the FCCm lattice structures for a given relative density range. This surface is a result of two subsequent nonlinear regression fits, which were obtained by means of the Curve Fitting Toolbox of MATLAB (2020a, MathWorks, USA). Firstly, an S–N curve was generated for each tested relative density with the form of Eq. (4).

4 $$\begin{aligned} S = C_1 N^{C_2} \end{aligned}$$

Once $C_1$ and $C_2$ values were obtained for the three batches, those values were used to fit two different exponential curves, one for each variable. Hence, the relative density is explicitly introduced as the function variable to define $C_1$ and $C_2$. In this case an offset was included in order to increase the flexibility of the variables.

5 $$\begin{aligned} C_1 = a \rho _{rel}^b + c \text {,} \; \; \; C_2 = d \rho _{rel}^e + f \end{aligned}$$

By introducing the expressions of Eq. (5) into Eq. (4), the next equation is obtained:

6 $$\begin{aligned} S = C_1 N^{C_2} = \left( a \rho _{rel}^{b} + c \right) N ^{\left( d \rho _{rel}^e + f\right) } \end{aligned}$$

Equation (6) describes the S–N–$\rho _{rel}$ surface that contains the fatigue life of the FCCm lattices, explicitly including the relative density in the equation of S–N curves.

## Results and discussion

### Manufacturing

The relative density of the scaffolds and their strut density are given in Table 1. For all the manufactured samples, the measured relative densities were always higher than designed, with the deviations between 2.39 and 7.72%.

The internal porosity of the struts is significantly lower in the RD01 specimens compared to the other samples. Even for the HIPed samples RD02 and RD03, the strut porosity is similar to as-built samples AB, indicating that the HIP process could not close the internal pores of the struts.

**Table 1:** Designed and manufactured relative densities.

	RD01	RD02	RD03	AB
Designed relative density (%)	10	20	30	20
Manufactured relative density (%)	12.39 ± 0.34	27.72 ± 0.31	37.11 ± 0.27	26.59 ± 0.2
Strut density (%)	99.57± 0.10	97.28 ± 0.18	97.18 ± 0.46	97.52 ± 0.16

### Quasi-static compression

The values obtained from the quasi-static compression tests are given in Table 2, including the standard deviation.

**Table 2:** Experimental values of tested FCCm lattice structures.

	Quasi-elastic radient (GPa)	0.2% offset stress (MPa)	Strain at stress offset (%)	Maximum stress (MPa)	Strain at maximum stress (%)
RD01	0.75 ± 0.01	17.95 ± 1.32	2.78 ± 0.25	23.94 ± 0.84	6.84 ± 0.32
RD02	4.96 ± 0.41	100.09 ± 0.35	3.15 ± 0.16	124.00 ± 0.65	7.77 ± 0.48
RD03	9.07 ± 0.60	181.79 ± 2.99	2.87 ± 0.05	234.43 ± 2.46	11.23 ± 0.53
AB	5.38 ± 0.39	121.00 ± 0.79	3.64 ± 0.23	138.40 ± 0.44	5.29 ± 0.29

#### Effect of relative density

**Figure 4:**
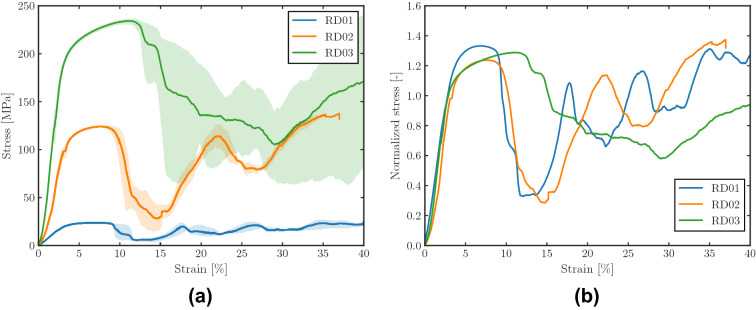
Stress strain curves of FCCm structures with different relative densities for (**a**) macroscopic stress and (**b**) normalized macroscopic stress.

Figure 4a shows the stress–strain curves of the HIP treated FCCm samples for different relative densities. The shaded area corresponds to the 95% confidence interval, which was obtained by combining the curves of the tested samples in each batch.

The scatter of the curves is very low in the elastic and beginning of plastic regions, and it increases in the stiffness drop region and afterwards. Moreover, even if the variability of the curves is rather small for RD01 and RD02, in the case of RD03 the curves of the samples show important differences, which leads to the wide shaded area of 95% confidence interval.

The fluctuation of the stress-strain curves varies depending on the relative density, and denser structures show a lower number of peaks and valleys for the same compression level of the sample. This indicates that the higher relative density favors a more homogeneous behavior of the structures, which do not collapse layer by layer or strut by strut, but more as a compact material. This effect can be better observed in Fig. 4b, which depicts the stress-strain curves normalized by the macroscopic yield strength of each of the structures. The figure also indicates that the stiffness drop of RD03 occurs at higher strain levels, and is more progressive than for RD01 and RD02.

**Figure 5:**
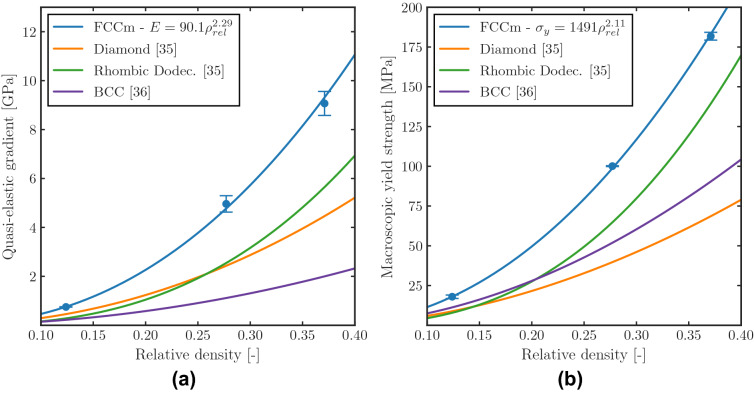
Gibson Ashby curves of FCCm and other bending dominated unit cells made of Ti6Al4V obtained from^35^ and^36^.

The stiffness and strength of the structures increase exponentially with the relative density, and the Gibson–Ashby curves of the FCCm lattices exhibit the typical bending dominated behavior. Figure 5 depicts the quasi-elastic gradient and macroscopic yield strength of the FCCm lattice structure and other bending dominated topologies under a wide range of relative densities, and the FCCm denotes superior stiffness and strength for the analyzed cases. These results are limited to a single load orientation, and can be explained by the different angle of the struts with respect to the load in the case of the FCCm lattice.

#### Effect of heat treatment

**Figure 6:**
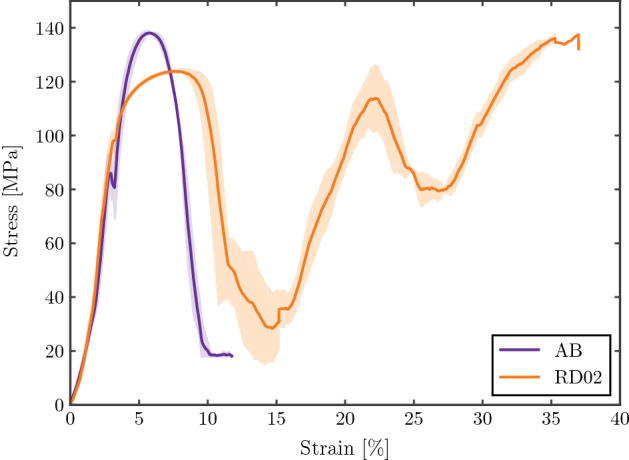
Stress strain curve of AB and RD02 structures.

The HIP process has a strong influence on the structures’ mechanical properties. As reported in several studies, HIP treatment of LPBF Ti6Al4V samples leads to residual stress relief, potential material porosity reduction and also a transition from the more brittle martensitic $\alpha '$ material structure to a more ductile $\alpha -\beta$ structure^21,26^. Figure 6 shows the difference between AB and HIP conditions. The AB samples have higher maximum stress, and a slightly higher quasi-elastic gradient. Nonetheless, the fracture of the structure is brittle, and occurs at lower strains compared to the samples after HIP treatment.

**Figure 7:**
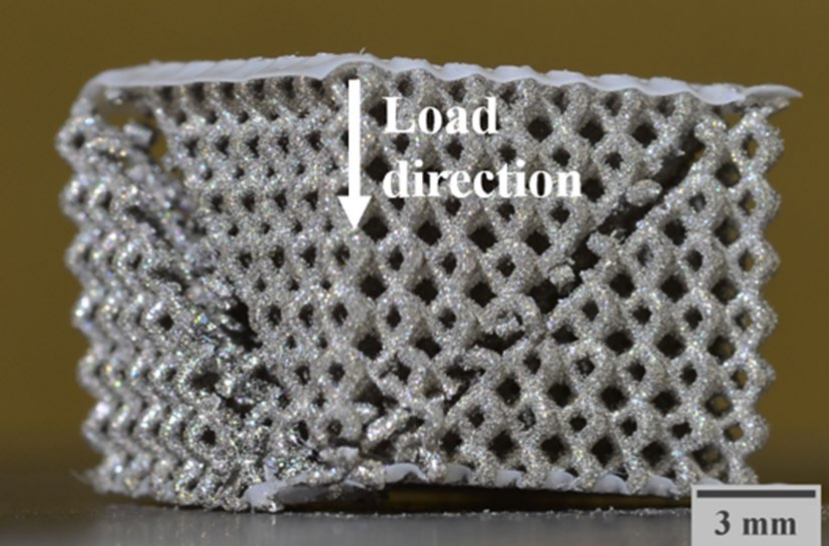
Fracture plane of the AB structure under compression load in [001].

After the first stress drop, the HIPed structure is able to continue absorbing energy, while for the AB structure, the fracture plane divides the structure in several parts with the consequent loss of the structural integrity needed to carry load and absorb energy as shown in Fig. 7. The difference in geometry between both structures may also play a role in this behavior, since the AB samples have fewer unit cells in each direction, making the structure more unstable after the slip plane appears. Figure 7 also shows that the slip plane corresponds to the [111] orientation, which is a common property of the FCCm unit cell regardless the relative density and heat treatment, under uniaxial load in [001] direction.

### Numerical simulation

The quasi-static mechanical properties of the numerical models are given in Table 3. The numerical model is capable of predicting the strength of the FCCm structures consistently, with a maximum relative error of 12% in the case of the RD01 structure. Nevertheless, the quasi-elastic gradients of the numerical models are far from the experimental values obtained. It is common to have significant differences between numerically and experimentally obtained mechanical properties of lattice structures, since numerical models neglect many imperfections that reduce the stiffness of lattices, and can even cause changes in their anisotropy^38,39^. These imperfections include the dross formation, the waviness of struts, the surface roughness, internal porosity, among others, and were not considered in the numerical models.

**Table 3:** Designed and manufactured relative densities.

	RD01		RD02		RD03	
	Exp.	FE	Exp.	FE	Exp.	FE
Quasi-elastic gradient (GPa)	0.75	1.54	4.96	10.45	9.07	20.89
0.2% offset stress (MPa)	17.96	20.16	100.09	98.5	181.79	174.88

#### Yield stress of structure and design criteria

The 0.2% offset stress is a widely used stress criterion to assess the strength of lattice structures, and often referred to as yield strength^11,35^. Nevertheless, this criterion should be used with caution if applied to lattice structures. Whereas the yield strength usually establishes the stress limit above which plastic deformation of the material begins, for lattice structures high plastic deformations may occur in large regions before reaching the macroscopic yield strength. This phenomenon is caused by the non-uniform stress fields that arise as the scaffolds are deformed.

**Figure 8:**
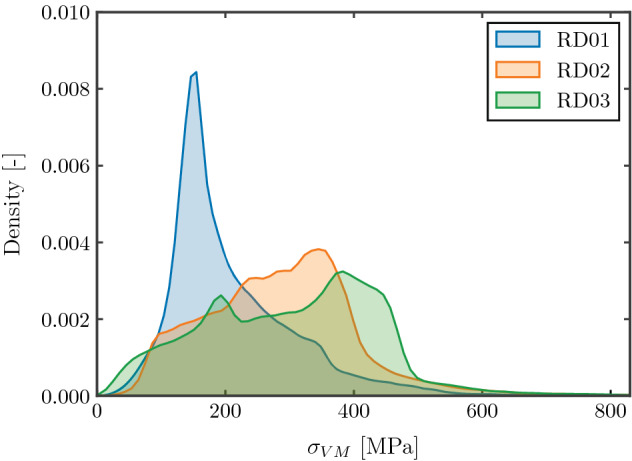
Probability distribution of Von Mises stress of the numerical models under same normalized macroscopic stresses.

Figure 8 depicts the distribution of the Von Mises stress for the numerical models corresponding to the tested specimens. The macroscopic stress applied to each model corresponds to the half of the macroscopic yield strength, obtained as 0.2% offset stress. The Von Mises stress corresponds to the actual stress of the bulk material within the structure, that is, the mesoscopic stress. The effect of relative density can be observed in the different shape of the distributions. For low relative densities, the proportion of low stress regions is much larger. The higher slenderness of the struts results in a more beam like load distribution, with large regions of the struts under low stress levels. As the relative density increases, the probability distribution covers a wider span of the stresses, and a larger proportion of the structure withstands higher stress levels for the same proportional macroscopic stress. The exponential growth of the Gibson Ashby curves can be explained by this evolution of the load carrying mechanism.

**Figure 9:**
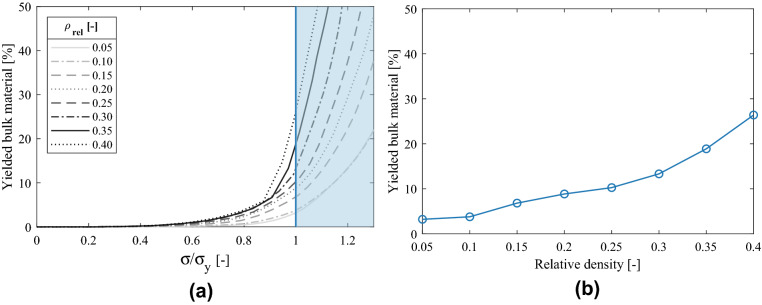
(**a**) Evolution of bulk material yielding for different relative densities, (**b**) proportion of yielded bulk material at macroscopic 0.2% offset yield stress for different relative densities.

The different stress distributions have significant implications when considering design criteria based on macroscopic stresses, such as macroscopic yield strength. If the stress state of the numerical models is analyzed for different macroscopic stress levels and different relative densities, it can be observed that the proportion of plastic deformation that occurs before the macroscopic yield strength is highly dependent on relative density, as indicated in Fig. 9a. The Figure depicts the percentage of the bulk material of the lattice structures above yield stress, and the ratio between the applied macroscopic stress and the macroscopic yield strength. $\sigma / \sigma _y = 1$ corresponds to the 0.2% offset stress, considered the macroscopic yield stress for each model, after which macroscopic plastic deformation is assumed (blue area in Fig. 9a).

Figure 9b indicates the percentage of the yielded volume of each structure at the macroscopic yield stress level corresponding to the 0.2% offset stress. As the relative density increases, the proportion of regions with plastic deformation is larger at the macroscopic yield stress level. For the FCCm lattice structures with relative density of 40%, above one quarter of the structure is undergoing plastic deformation before reaching the macroscopic yield criterion.

This information should be considered when designing parts that include lattice structures in case the bulk material is supposed to work only within the elastic region. Furthermore, the irregularities of the surface and manufacturing deviations were not accounted for in these simulations. These effects increase the stress level of the lattice structures, thus enhancing the probability to develop plastic strains before expected in the design process.

### Fatigue properties

#### Global S–N curves

**Figure 10:**
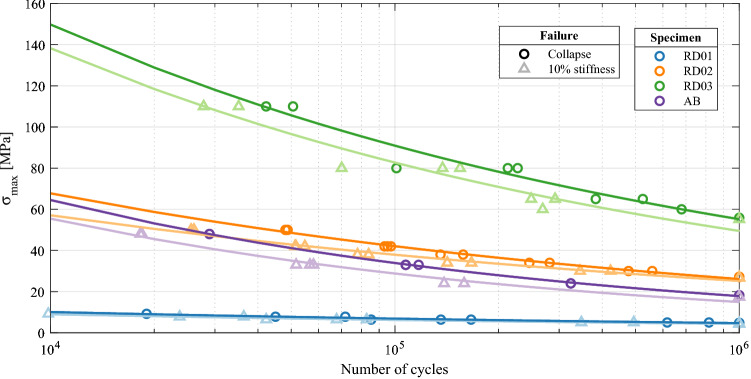
S–N curves of FCCm lattice structures for two different failure criteria.

Figure 10 depicts the S–N curves obtained from the experiments, including experimental data obtained using both the failure criteria of 10% stiffness drop and collapse of the structure. The experimental points were adjusted to Basquin’s exponential curves $S=C_1 N^{C_2}$ and the obtained coefficients are given in Table 4.

The variation of the relative density comprises a very wide range of fatigue resistance values for the FCCm structure, across different orders of magnitude. This variability is suitable in order to tailor the mechanical properties of the structure considering the required fatigue performance under different load cases, or to design graded lattice structures with fatigue strength constraints.

As expected, the HIP process significantly improves the fatigue life of the structures, even if in this case the HIP process was not able to close the internal pores of the struts. Therefore, the change in microstructure can be considered as the responsible for fatigue life enhancement^21^.

**Table 4:** Coefficients for Basquin’s exponential S–N curves of FCCm structures.

	10% stiffness loss		Collapse failure	
	$C_1$	$C_2$	$C_1$	$C_2$
RD01	39.07	−0.16	46.24	−0.17
RD02	293.19	−0.18	455.32	−0.21
RD03	1078.70	−0.22	1102.60	−0.22
AB	761.35	−0.28	843.60	−0.28

#### Evolution of stiffness and failure criteria

**Figure 11:**
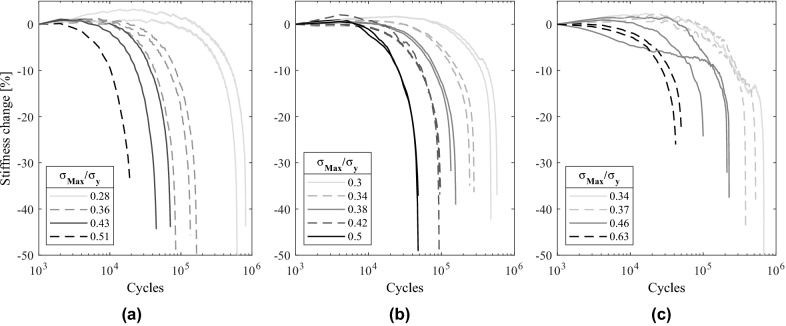
Evolution of stiffness under different loads and relative densities of (**a**) 10%, (**b**) 20% and (**c**) 30%.

The loss of stiffness of lattice structures during fatigue loading is a progressive degradation process in which the damage, the failure or the local plastic deformation of each strut gradually reduces the macroscopic stiffness of the structure. This process is depicted in Fig. 11 for the different relative densities of FCCm lattice structures. The legends include the proportion of maximum stress of the cycles with respect to the macroscopic yield strength of the structures.

The damage that results in stiffness loss is accumulated at different rates depending on the load and the relative density. The highest stress levels induce more pronounced stiffness losses from the beginning of the test, with small regions of stable stiffness. Nonetheless, lower stress levels result in large stable stiffness regions before the accumulation of damage begins. This trend is observed for all the analyzed specimens, however, the rate of damage accumulation and its stability also depends on the relative density.

**Figure 12:**
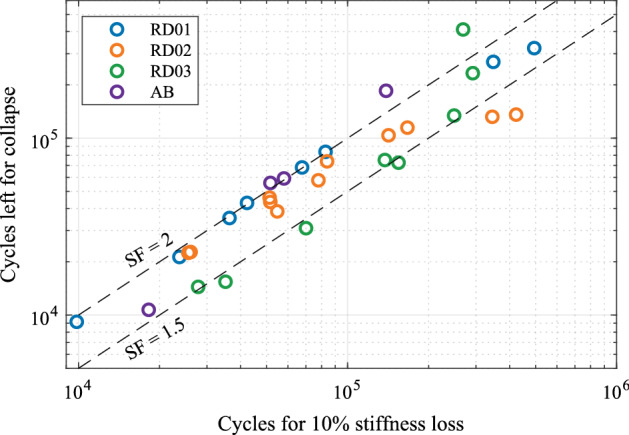
Cycles left until collapse for FCCm structures with different relative densities depending on cycles at 10% stiffness loss.

Establishing a fatigue criteria based on the 10% stiffness loss of the structure allows to guarantee the integrity of the lattice structure, as well as it hinders the excessive loading of parts adjacent to the lattice structure in load bearing applications. Nonetheless, it is important to notice that stiffness loss criteria for lattice structures imply different safety factors (SF) depending on the relative density. SF is defined by the ratio between the cycles until collapse and the cycles elapsed until the established fatigue criterion. Figure 12 presents the relationship between the elapsed cycles at the 10% stiffness loss, and the cycles left until total collapse of the structure, for each of the tested specimens. The two dashed lines in the figure correspond to SF = 2 (y = x line) and to SF = 1.5 (y = 0.5x line) respectively.

In low cycle fatigue (LCF) there is a correlation between relative density and SF: the RD01 specimens coincide with the SF = 2 line, the RD03 is in good agreement with the SF = 1.5 line, and RD02 is between both lines, which means that a higher relative density implies a lower SF. It must be noted that the tendency of RD02 is not linear and presents higher variability with respect to the stress level compared to RD01 and RD03. On the other hand, for high cycle fatigue (HCF) the SF of RD01 and RD02 decreases with respect to LCF, whereas for RD03 the SF increases. The increase for RD03 samples above SF = 1.5 line can be explained by the tendency change of the stiffness decrease as depicted in Fig. 11c for the lowest stress levels. For the AB samples, the SF is similar to the HIPed samples, nevertheless, the decrease of stress level tends to increase the SF, which is a tendency contrary to the one observed in the RD02 samples.

The ratio between the SF and stiffness loss enables the prediction of cycles left for FCCm lattice structures and thus a safer integration in load bearing applications. Furthermore, instead of establishing a certain stiffness drop as a failure criterion, it is also possible to use a certain SF as failure criterion, which can be implemented by calculating the stiffness drop corresponding to each relative density and stress level.

Strain accumulation must also be considered to consider the failure of the structure at 10% stiffness loss. For all the tested stress levels, the mean accumulated strain at 10% stiffness drop was 0.36%, 0.39% and 0.32% for RD01, RD02 and RD03 structures respectively, which ensures structural stability.

#### Normalization of S–N curves

**Figure 13:**
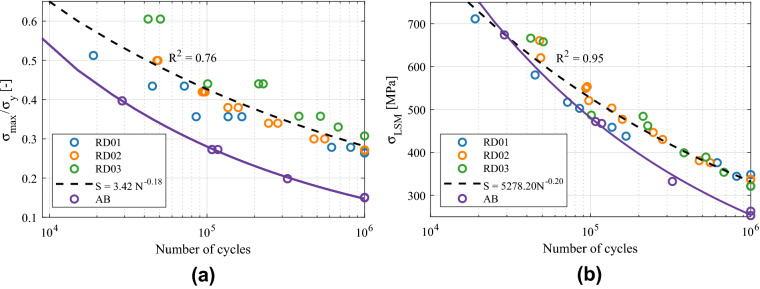
S–N curves of collapse of the structures normalized by (**a**) yield strength and (**b**) Local Stress Method.

Figure 13 shows the normalized S–N curves of the FCCm structures based on the final collapse failure criterion. Figure 13a depicts the S–N curves normalized with the macroscopic yield strength of each structure for different relative densities, and the fitted dashed line corresponds to all the HIPed samples. A clear difference can be observed between the studied relative densities, and the normalized fatigue strength increases with the relative density, which limits the fatigue prediction capability of this method. The effect of heat treatment can be observed with the AB curve below all the normalized curves after HIP, regardless the relative density.

It is common to normalize the S–N curves by dividing the stress with a quasi-static macroscopic property of the lattice structure being analyzed, e.g. the macroscopic yield strength or plateau stress^20,40^. The advantage of this approach is that very different factors such as the variation of relative density, microstructure, or manufacturing defects can be accounted in an implicit manner, thus obtaining curves that enable the prediction of fatigue life. Nonetheless, the accuracy of this approach is variable across different works and studied unit cell types^20,41,42^, and in the case of the FCCm unit cell it has only a limited reliability.

The normalization based on local tensile stresses of the beam models is given in Fig. 13b, and it shows a very good agreement for all experimental data corresponding to HIPed specimens, regardless the relative density of the structures. The level of accuracy is higher in HCF, presumably because the assumption of elastic behavior is more valid in this region. The effect of heat treatment can also be observed in Fig. 13b, indicating that the difference between AB and HIP increases from LCF to HCF.

It is worth noting that an analytical beam model considering the tensile stresses exhibits a higher accuracy than the normalization with the macroscopic yield strength, despite the latter implicitly considers more variables. This might be explained by the fact that the macroscopic yield of lattice structures is a compression driven phenomenon under macroscopic compression, while the fracture of struts and progressive damage accumulation is induced mainly by tensile stresses developed across each strut under compressive loads.

**Figure 14:**
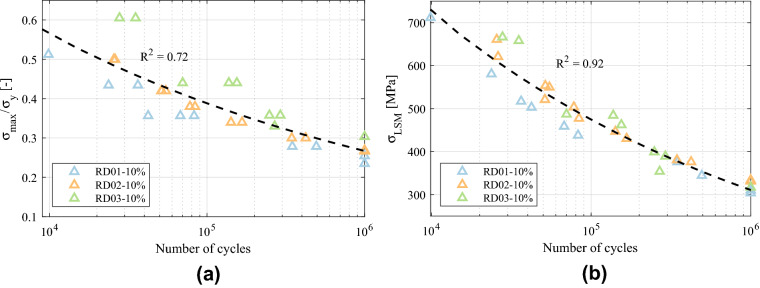
S–N curves of 10% stiffness loss criterion normalized by (**a**) yield strength and (**b**) Local Stress Method.

On the other hand, these methods to predict fatigue life are not as accurate if 10% stiffness loss criterion is used, as depicted in Fig. 14. In the case of yield strength normalization, the $R^2$ is even lower than for collapse, and three different tendencies can be distinguished. Local Stress Method fits better, but the accuracy also decreases compared to Fig. 13b. This loss of accuracy arises because of the fact that a prescribed stiffness loss corresponds to different SF for each relative density and stress level. Nonetheless, for a prescribed SF the normalization accuracy corresponds to the one in Fig. 13. The fatigue failure surface was developed in order to have more flexibility in the prediction of fatigue life with various failure criteria.

#### Fatigue failure surface

The constants resulting from the exponential fits for fatigue failure surface are given in Table 5. For each of the considered failure criteria a failure surface can be defined by introducing the constants in Eq. (6). The differences in sign for some constants for different failure criteria arise from the very different behavior of the evolution of stiffness after the 10% loss for each structure. This method provides an accurate tool ($R^2=0.99$ and $R^2=0.98$ for 10% stiffness loss and collapse criteria, respectively) to predict the fatigue life of FCCm lattice structures within a wide range of relative densities.

**Table 5:** Constants from exponential fits for stiffness loss and collapse fatigue failure surfaces.

	10% stiffness loss	Collapse
a	118600	22940
b	4.77	3.07
c	33.51	8.53
d	−4.07	0.02
e	4.19	−0.74
f	−0.16	−0.26

**Figure 15:**
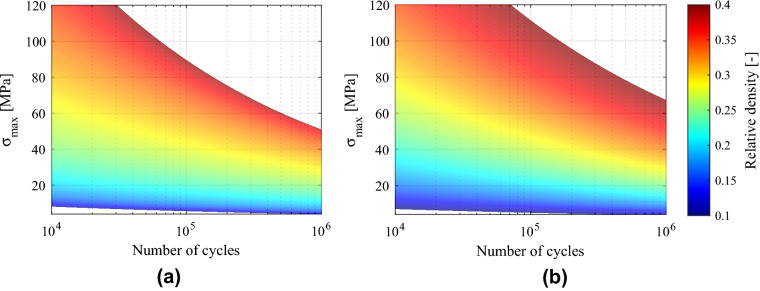
Failure surface after HIP for (**a**) 10% stiffness loss and (**b**) collapse of structure.

The failure surface of Fig. 15a corresponds to the 10% stiffness loss of the structures, while the Fig. 15b depicts the collapse of the structures for each relative density. As expected, the area corresponding to the 10% stiffness loss is smaller than for the collapse, since the former criterion is more restrictive.

The failure surface is a highly flexible tool to predict failure of lattice structures under several fatigue criteria. In this work the 10% stiffness loss was used apart from the collapse of the structure, but adopting any stiffness based criteria is also possible. Furthermore, this method also allows considering different admissible stiffness loss values depending on the relative density: for lower relative densities, higher stiffness losses are admissible before collapse (Fig. 12).

On the other hand, for rationally designed and multifunctional lattice structures that are being developed^43,44^ to achieve specific mechanical behaviors, the fatigue life is not necessarily proportional to quasi-static mechanical properties. This is also the case of auxetic metamaterials^42^, and for these structures the fatigue failure surface can be a useful predictive tool.

#### Fatigue strength at $10^6$ cycles

**Figure 16:**
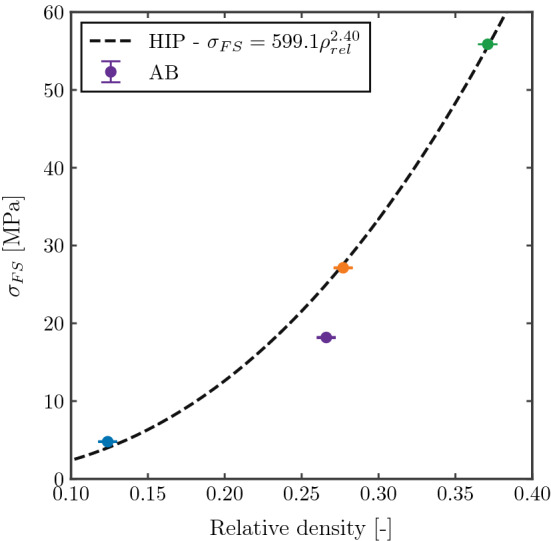
Fatigue strength at $10^6$ cycles of FCCm structures for different relative densities and post treatments.

The resulting FS at $10^6$ cycles for each of the tested structures is given in Fig. 16. As expected, the fatigue strength varies with the relative density following an exponential curve as the quasi-static mechanical properties listed in Table 2. Moreover, the HIP treatment increases the FS of the structures as expected.

**Figure 17:**
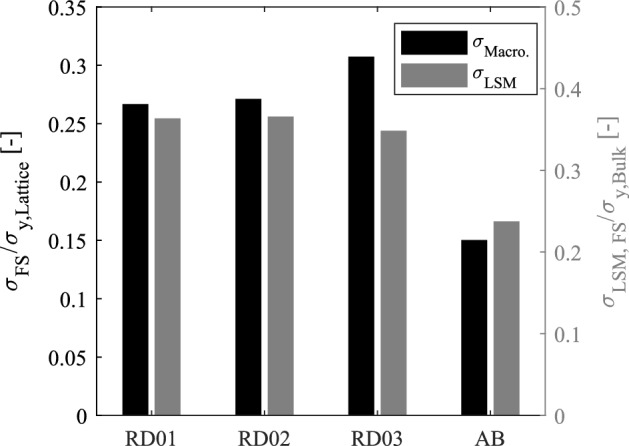
Normalization of fatigue strength with yield strength and local stress method.

Figure 17 indicates the FS of each structure after normalization with macroscopic yield strength, and using the LSM normalized by the yield strength of the bulk material. The normalized fatigue strength of HIPed samples increases slightly with relative density, from 0.27 to 0.31, while the AB sample has a normalized FS of 0.15. The FS at $10^6$ for several bending dominated structures is reported to lay between 0.15 and 0.24 for AB condition^45^. The higher normalized FS for HIPed structures is in line with results reported in literature^46,47^, and the LSM also indicates that HIP treatment increases the FS of the structures, regardless the relative density. This can be mainly attributed to the $\alpha + \beta$ microstructure of the HIPed samples compared to the $\alpha '$ of the AB condition.

It should be mentioned that the whole work was focused on the macroscopic properties of the lattice structures. In a future work, it would be interesting to analyze the fracture process of the structures under fatigue. It would be also interesting to study whether titanium alloys that achieve a non-layer-wise fracture in compression tests^37^ could improve the fatigue life of these structures.

## Conclusions

The relative density of the lattice structures is one of the most relevant parameters that enables the control and design of their mechanical properties. This study analyzed the quasi-static and dynamic mechanical properties of lattice structures based on the FCCm unit cell with different relative densities, and the interaction between macroscopic and mesoscopic variables. The main conclusions are as follows:

- The quasi-static mechanical properties of FCCm lattice structures follow the expected exponential curve, and their quasi-elastic gradient and yield strength are above the ones of other bending dominated unit cells in the tested direction.
- Macroscopic 0.2% offset stress does not represent the transition from elastic to plastic deformation of the bulk material for lattice structures. In fact, the relative volume of the bulk material undergoing plastic deformation at macroscopic 0.2% offset stress varies with the relative density.
- The S–N curves of the FCCm unit cell made of HIPed Ti6Al4V were obtained under compressive load and R=10 for different relative densities.
- The Local Stress Method, which has proved valuable for diamond lattices, is also able to predict the fatigue life of FCCm structures within a very wide range of relative densities.
- A stiffness based fatigue failure criterion is presented to ensure structural integrity and load carrying capacity, showing that the Safety Factor depends on the relative density of the structures as well as on stress level.
- The developed fatigue failure surface method accurately describes the fatigue life of FCCm lattices, and its flexibility enables the use of stiffness based fatigue failure criteria.
